# Multi-Grain Rice Diet Decreases Risk of Breast Cancer in Korean Women: Results from the Health Examinees Study

**DOI:** 10.3390/nu12082273

**Published:** 2020-07-29

**Authors:** Woo-Kyoung Shin, Hwi-Won Lee, Aesun Shin, Jong-koo Lee, Sang-Ah Lee, Jung Eun Lee, Daehee Kang

**Affiliations:** 1 Department of Preventive Medicine, Seoul National University College of Medicine, 103 Daehak-ro, Jongno-gu, Seoul 03080, Korea; shiningwk@gmail.com (W.-K.S.); hwiwon@snu.ac.kr (H.-W.L.); shinaesun@snu.ac.kr (A.S.); 2 Department of Biomedical Sciences, Seoul National University Graduate School, Seoul 03080, Korea; 3 Cancer Research Institute, Seoul National University, Seoul 03080, Korea; 4 Department of Family Medicine, Seoul National University Hospital, Seoul 03080, Korea; kcdc7000@gmail.com; 5 Department of Preventive Medicine, Kangwon National University School of Medicine, Chuncheon-si, Gangwon-do 24341, Korea; sangahlee@kangwon.ac.kr; 6 Department of Food and Nutrition, Seoul National University, Seoul 08826, Korea; jungelee@snu.ac.kr; 7 Research Institute of Human Ecology, Seoul National University, Seoul 08826, Korea

**Keywords:** dietary patterns, multi-grain rice, breast cancer risk, Korean women, the Health Examinees (HEXA) study

## Abstract

Although a number of studies explain the association between dietary patterns, which take into account that foods are eaten in combination, and breast cancer risk, the findings are inconsistent. We examined the association between dietary patterns and multi-grain rice intake, and the risk of breast cancer in a large-scale prospective cohort study in Korean women. A total of 93,306 women aged 40–69 years from the Health Examinees-Gem (HEXA-G) study (2004 and 2013) were included. We obtained Information on cancer diagnosis via linkage to the Korea Central Cancer Registry. Factor analysis was conducted to obtain dietary patterns, and Cox proportional models were used to estimate the hazard ratios (HRs) and 95% confidence intervals (95% CI) for breast cancer risk. For 494,490 person-years, 359 new cases of breast cancer occurred. We identified three major dietary patterns, that explained 23.9% of the total variance based on daily total food intake (g/day) from 37 food groups: the meat dietary pattern (higher intake of bread and red meat), the white rice dietary pattern (higher intake of white rice and lower intake of multi-grain rice), and the other pattern. Women who had higher white rice dietary pattern scores had a 35% higher risk of breast cancer, than did women with lower white rice dietary pattern scores (multivariable HR 1.35; 95% CI 1.00–1.84 for the highest vs. lowest quartile of the white rice dietary pattern scores, p for trend = 0.0384). We found that women who consumed three or more servings of multi-grain rice per day had 33% lower risk of breast cancer than did those who consumed one or less multi-grain rice serving per day among women under 50 years of age (multivariable HR 0.67; 95% CI 0.45–0.99, p for trend = 0.0204). Our study suggests that a multi-grain rice diet may be associated with lower risk of breast cancer in Korean women.

## 1. Introduction

The incidence rate of breast cancer varies widely worldwide [1]. In Korea, the incidence and mortality of breast cancer have increased consistently, however the 5-year relative survival rates for breast cancer was 92.7% in 2016 [2]. Therefore, there is growing interest in the potential effect of modifiable factors on breast cancer risk.

Several epidemiologic studies have linked the risk of breast cancer with individual foods and nutrients such as fruits and vegetables [3], fats [4], red meat [5], milk [6], fish [7], and dietary supplements containing soy isoflavone [8], with inconsistent results [9]. However, foods and nutrients are consumed in combination rather than separately, and the components in these foods may have a synergistic effect on breast cancer risk [10]. Exploration of dietary patterns that potentially reflect possible foods and nutrients working together may overcome the limitation of assessments of individual food items [11].

Several epidemiological studies have investigated the association between dietary patterns and breast cancer risk. Previous cohort studies indicated that those who had dietary patterns, characterized by high intake of fruit and vegetables [12,13], fish, poultry, and dairy products [14] had reduced risk of breast cancer. Increased breast cancer risk has been associated with a Western dietary pattern that is high in red and processed meat, refined grains, sweets and desserts [15], French fries, and pasta [16], as well as a drinker dietary pattern [17].

Asian dietary habit differ from those of western populations; Asian populations consume much less meat and more carbohydrate compared to the western populations [18,19]. However, most of the prospective studies on dietary patterns and breast cancer risk have been conducted in Western countries, and few studies regarding dietary patterns associated with breast cancer risk have been conducted in Asian populations [20,21]. Given that dietary patterns vary among populations because of different dietary habits, preferences and availability [11], further studies are needed regarding the prevention of breast cancer in the Korean population. To our knowledge, only one case–control study reported that the vegetable-seafood pattern decreased the risk of breast cancer [22], and no prospective cohort study has been undertaken in Korean women.

In this study, we aim to estimate the effect of dietary patterns and multi-grain rice intake on the risk of breast cancer development by using a large-scale prospective cohort study of Korean women between 2004 and 2013.

## 2. Method

### 2.1. Study Population

This study used baseline data from the Health Examinees-Gem (HEXA-G) study, which was derived from the HEXA study [23,24]. The HEXA study is a community-based large-scale prospective cohort study composed of Korean adults aged 40–69 years who were recruited from health examination centres from 2004–2013. At a baseline, a total of 173,342 men and women were recruited.

Updated from the previously published HEXA studies [23,24], the HEXA-G study applied additional eligibility criteria at the participating sites. The criteria precludes sites that only participated in the pilot study from 2004 to 2006, had less than 2 years of follow-up, or did not meet quality control criteria for bio specimen [25]. In the HEXA-G study, a total of 93,306 women remained. Among the participants, we excluded those who had no information regarding the date and cause diagnosis of cancer from the Korea Central Cancer Registry (*n* = 8,531). Participants were also excluded if they did not answer the Food Frequency Questionnaires (FFQs) (*n* = 1,165) and had insufficient values (<500 or >3500 kcal/day) for total energy intake (*n* = 1,069); 4000 participants who already had any type of cancer at baseline were excluded. We also excluded women who were diagnosed with breast cancer during the first 2 years of the follow-up period to lessen the impact of reverse causality (*n* = 221). Finally, we considered 78,320 women in this analysis.

The HEXA study is a component of the Korean Genome and Epidemiology Study (KoGES). The study protocol was approved by the Institutional Review Board (IRB) of the Seoul National University Hospital (IRB number 0608-018-179) and the Ethics Committee of the KoGES of the Korea National Institute of Health (IRB number 2014-08-02-3C-A).

### 2.2. Assessment of Dietary Intake

Usual dietary intake was assessed using a validated semi-quantitative FFQ during the previous one year [26]. Interviewers asked participants how often they had consumed 106 foods during the previous one year. The frequencies were classified into nine levels from never or seldom to three times or more a day. The portion size was divided into three levels depending on the average size of each food item. Component food items of the white rice dietary pattern included in the FFQ items included white rice (1 serving, 220 g) and multi-grain rice (1 serving, 220g) [27]. The usual intake of each food item was converted to daily intake based on the reported frequency and amount of each food item. The daily total calories and nutrient intakes were calculated using a Korean standard food composition table [28].

### 2.3. Identification of Breast Cancer and follow-up of the Cohort

Information on the date of initial diagnosis and cause of cancer was ascertained by linkages to the Korea Central Cancer Registry, until 31 December 2014. Participants gave written informed consent for secondary data linkages, such as the Korean Central Cancer Registry. Cancer incidence was defined based on the 10th revision of International Classification of Disease (ICD-10) as follows: cancer incidence (C), and breast cancer incidence (C50).

### 2.4. Collection of Sociodemographic and Lifestyle Information

Each participant’s age, educational level (uneducated, elementary school, middle school, high school, college, undergraduate, graduate or more), regular exercise (no, yes), alcohol intake (never, former, current), smoking status (never, former, current), marital status (married, cohabiting, unmarried, divorced, widowed), menopausal status (no, yes), the number of parity, age at first birth, age at menarche, oral contraceptive use (no, yes), and the presence of family history of cancer (no, yes) were obtained using the structured questionnaire. We obtained information on the participant’s height (cm) and weight (kg) directly measured by the medical staff. Body mass index (BMI, kg/m^2^) was calculated by dividing body weight (in kilograms) by the square of height (in metres).

### 2.5. Statistical Analysis

We grouped a total of 106 food items into 37 food groups according to the similarities of their nutrient profiles based on previous studies [29,30,31] (Table 1). Next, we combined the intake amount for each food group per participant and calculated the daily amount (g/day) of each food group.

Factor analysis (principal components) was used to derive dietary patterns based on the 37 food groups in our analysis. The factors were rotated using an orthogonal transformation to simplify the factor structure for easier interpretation. The more involved a variable was, the higher the weight. Variables unrelated to a dietary pattern would be weighted close to zero. The factor score for each dietary pattern was calculated by summing the intakes of all the food groups weighted by their factor loadings, and each participant was assigned a factor score for each dietary pattern [12]. A higher factor score indicated a higher level of adherence to the dietary pattern. Food groups with factor loading values ≥ |0.2| were defined as high contributors to each dietary pattern and were used to characterize each dietary pattern. We determined three factors based on a combination of eigenvalues, the Scree test, and our interpretations of the derived factors [15]. We labelled these three patterns the ‘meat dietary pattern’, the ‘white rice dietary pattern’, and the ‘other pattern’. Participants were divided into four categories based on quartiles of factor scores for each dietary pattern.

Cox proportional hazards models were fit to assess the relationship between each dietary pattern and the incidence of breast cancer; age was used as the time scale in Cox proportional hazard models. Age at baseline was used as the entry time to reflect the left-truncation time of the data and age at cancer diagnosis or final follow-up was the exit time. Person-years of follow-up were estimated from the date of study entry until the date of breast cancer diagnosis, date of any cancer diagnosis, or the end of the follow-up period (31 December, 2014). In separate analyses, we also assessed the relationship between each individual component of the white rice dietary pattern and the risk of breast cancer. Participants were classified into three categories based on the distribution of their reported daily intake of each component of the dietary pattern. Because the number of participants who never or rarely consumed white rice (83%) were more than those who never or rarely consumed multi-grain rice (43%), we categorized these women into <1 serving/day, 1–2 serving/day, or ≥3 servings/day for white rice consumption and ≤1 serving/day, 2 serving/day, or ≥3 servings/day for multi-grain rice consumption groups.

Multivariable hazard ratios (HRs) were adjusted for BMI (kg/m^2^, continuous), total energy intake (kcal/day, continuous), educational level (middle school or less, high school or college, undergraduate or more), parity (nulliparous, 1, 2, more than 3), age at first birth (nulliparous, <25, ≥25 years), age at menarche (<15, 15, ≥16 years), regular exercise (no, yes), alcohol intake (never, ever), and the presence of a family history of breast cancer (no, yes). Tests of linear trend were calculated by assigning the medians to each category and evaluated by the Wald test.

If we had missing variables (less than 3% of each variable), we assigned the median value or most common category to missing variables in this study. SAS software version 9.4 (SAS Institute, Cary, NC, USA) was used for all analyses. All p-values were two-sided tests, and statistical significance was set at *p* ≤ 0.05.

## 3. Results

We identified three major dietary patterns in the final models, ‘meat dietary pattern’, and ‘white rice dietary pattern’, and the ‘other pattern. Three dietary patterns, which explained 23.9% of the variance in the 37 food groups in our factor analysis. The factor loading matrix for the three patterns, for which a positive loading indicates a positive association between the dietary pattern and the food group and a negative loading indicates an inverse association, is shown in Table 2. The meat dietary pattern explained 5.8%, the white rice dietary pattern explained 4.7%, and the other pattern explained 13.4% of the variance in food intake. The meat dietary pattern is characterized by a high levels of bread, pizza and hamburger, cereals and snacks, noodles, dumplings, red meat, red meat by-products, poultry, processed meat and fish. The white rice dietary pattern was characterized by higher intake of white rice and salt-fermented fish and lower intake of multi-grain rice, tubers, legumes and soybean paste, tofu, nuts, and milk. The other pattern is characterized by high loading of yellow-green vegetables, other vegetables, mushrooms, seaweed, lean fish, fatty fish, dried anchovy, legumes and soybean paste, and tofu.

For 494,490 person-years of follow-up (average follow-up: 6.3 years) for 78,320 women, a total of 359 breast cancer cases were newly diagnosed, and these cases were included in this analysis. The participants’ mean age, BMI, and total energy intake were 52.3 (years), 23.6 (kg/m^2^), and 1981.0 (kcal/day), respectively. We compared the general characteristics of the participants across quartiles of the factor score for each dietary pattern (Table 3). Women with higher meat dietary pattern scores were younger, more highly educated, more likely to be married or cohabitanting (not including being unmarried, divorced or widowed) and to have higher proportion of ever drinkers and ever smokers than those with lower scores. Women with higher white rice dietary pattern scores were younger, more obese, less educated, less likely to be married or cohabitanting, were less physically active, and had a higher proportion of ever drinkers and ever smokers than those who scored lower on this pattern. Women who scored high on the other pattern were older, more likely to be married or cohabitanting, and were more physically active than those who scored low on this pattern.

In addition, higher the meat dietary pattern scores and other pattern scores were associated with higher levels of total energy intake, whereas the white rice dietary pattern scores were associated with lower levels of total energy intake.

Multivariable HRs of breast cancer according to quartile category of each dietary pattern score are shown in Table 4. Women who had higher white rice dietary pattern scores tended to be associated with an increased risk of breast cancer; multivariable HR (95% CI) comparing the highest with the lowest quartile of the white rice dietary pattern scores was 1.35 (1.00, 1.84) (*p* for trend = 0.0384). In contrast, the meat dietary pattern and the other pattern were not associated with the risk of breast cancer.

Table 5 and Table 6 provide the associations between consumption of major components of the white rice dietary pattern, white rice and multi-grain rice, and the risk of breast cancer. We found an inverse dose–response relationship between the risk of breast cancer and multi-grain rice intake among women under 50 years of age. Women who consumed three or more servings of multi-grain rice per day had a 33% lower risk of breast cancerthan those who consumed one or less serving of multi-grain rice per day consumed among women under 50 years of age (HR: 0.67, 95% CI: 0.45–0.99, *p* for trend = 0.0204). However, we observed women who consumed three or more servings of white rice per day had no significantly increased risk of breast cancer (HR = 1.29, 95% CI = 0.94–1.76; *p* for trend = 0.1363) compared with women who almost never consumed white rice.

## 4. Discussion

In this large prospective cohort study, we identified three dietary patterns: the ‘meat dietary pattern’, the ‘white rice dietary pattern’, and the ‘other pattern’. We found that higher white rice dietary pattern scores were significantly associated with increased risk of breast cancer. However, there were no associations between the meat dietary pattern or the other pattern and breast cancer risk among women.

Previous prospective cohort studies on the association between dietary pattern and breast cancer risk conducted in western populations [14,15,17,32] and in Asian populations [21,33,34] have been reported; however, the results from these studies have been inconsistent. In the Nurse’s Health Study (NHS) [14,17] and the Swedish Mammography Screening Cohort study [15], there were no overall association between dietary patterns and breast cancer risk [14,17] and postmenopausal breast cancer risk [15]. However, the prudent diet, which is characterized by higher intake of fruits, vegetables, whole grains, low-fat dairy products, fish and poultry was inversely associated with estrogen receptor- negative breast cancer risk [15,32] and breast cancer risk in premenopausal women [32]. In contrast, the drinker dietary pattern (wine, beer, and spirits) had an increased the risk of breast cancer [17]. In a prospective cohort study of Japanese women, the western dietary pattern (bread, beef, processed meat, coffee, tea, soft drink, and sauce) is associated with an increased risk of breast cancer [35]; however, the animal product diet (meat, deep-fried foods, fried vegetables, fish pasted and salt-preserved fish) gave a decreased risk of breast cancer among premenopausal women [21]. A Singapore Chinese Health Study showed that there was a dose-dependent trend of decreasing breast cancer risk for the vegetable-fruit-soy dietary pattern among postmenopausal women [34].

In our study, we identified the white rice dietary pattern, which is characterized by high intake of white rice and low intake of multi-grain rice, in Korean women. Similarly, previous studies identified the white rice pattern, characterized by very high consumption of white rice and very low consumption of whole grain [31] and the traditional pattern which included high intake of white rice [36], in Korean women. One of the main patterns of Korean diet is the rice dietary pattern because rice is typically consumed as a staple food among Koreans [18].

This study found that the white rice dietary pattern was significantly associated with the risk of breast cancer. White rice is classified as a refined grain because during the refining process the outer layer of the grain containing bran and germ is striped out, and white rice that only contains the endosperm is produced [35]. A case-control studies showed a positive association between white rice consumption [37] or a high glycaemic index [38,39] and breast cancer risk. White rice consumption, which includes eating endosperm that is composed primarily of starch and that is known to have a high glycaemic index, is related to an increased breast cancer risk [39].

In this study, the main components of the white rice dietary pattern were white rice and multi-grain rice. Our results showed that higher consumption of multi-grain rice was associated with a lower risk of breast cancer among women under 50 years of age. In agreement with our findings, a cohort study [40] and case-control studies [37,41] reported a reduced breast cancer risk for high intake of whole-grain products. We found an inverse association between whole-grain intake and breast cancer risk only in women under 50 years old, the median age of menopause in Korean women [42], but not in the entire study population. Several characteristics for breast cancer can vary according to menopausal status because of the different hormonal environments [43].

Whole grains have more nutritional value than refined grains [44,45] because the refining process removes bran and germ containing many nutrients [46]. The rice in the multi-grain group showed features of whole grain [46]. Evidence supporting the protective effect of whole grain intake on the risk of breast cancer has been shown from previously studies. Whole grains are rich in dietary fibr, which have protective effects against cancer by means of increasing fecal bulk and possibly reducing the absorption of carcinogenic agents [45,47]. Dietary fiber can reduce breast cancer risk through binding estrogens in the colon and increasing the fecal excretion of estrogens, resulting in a reduction in estrogen concentrations [48]. A meta-analysis of 16 prospective studies found that dietary fibr intake was associated with a reduced risk of breast cancer [49]. Whole grains contain high amounts of antioxidants, including vitamins and trace minerals such as selenium and zinc [45]. Vitamin E plays protective roles against cancer by preventing carcinogen formation and blocking carcinogen-cell interactions [50]. In addition, whole grains are an important source of phytoestrogens including lignans. Lignans such as phytoestrogens might reduce breast cancer risk because lignans may have antiestrogenic effects [51], and phytoestrogens have antiproliferative properties in the breast, even though phytoestrogens have been shown to have significant estrogenic/antiestrogenic effects [52].

The strength of our study is that it is a prospective cohort study that eliminated potential for recall biases that can occur in either case-control studies or retrospective cohort studies. To the best of our knowledge, this is the first prospective study to examine the association between dietary patterns and breast cancer risk in Korean women. In addition, we used cancer diagnosis data that had high completeness approximately 97.8% in 2014 [53] and high sensitivity (81.2%) in breast cancer history [54] because the data on cancer diagnoses were retrieved from the Korea Central Cancer Registry.

Nevertheless, our study had some limitations. First, we assessed dietary intake only at baseline and could not control the possibility that some changes may have occurred in participants’ dietary habits may have occured during follow-up [55]. More accurate measurement tool for long-term diet are needed for follow-up. Second, we evaluated breast cancer risk without considering clinical factors, including mammographic breast density and hormone receptor status because we could not obtain information on clinical data for breast cancer patients. The association between dietary factors and breast cancer risk may vary depending on clinical factors such as hormone receptor status [56,57]. Further prospective studies that are able to use clinical data are needed to evaluate breast cancer risk. Third, the number of total participants in this cohort is fairly large and the duration of the follow-up period was not short, nevertheless, the number of breast cancer cases was limited, particularly in the subgroup analysis. Additionally, factor analysis depends on several subjective or arbitrary decisions regarding the selection of included variables, the number of retained factors, and the method of rotation that can have some impact on their interpretation [58]. However, factor analysis is a statistical tool for aggregating inter-related variables into composite factors that is a commonly used in other epidemiological studies. Finally, women under 50 years of age may have developed breast cancer after age 50, which could lead to misclassification of the age group. Therefore, we need to assess breast cancer risk considering the age at diagnosis of breast cancer in future studies. In conclusion, the multi-grain rice diet was associated with a reduced risk of breast cancer in Korean women under 50 years of age. Additional studies are needed to examine associations between diet and breast cancer risk considering breast cancer subtypes and the hormone receptor status.

## Figures and Tables

**Table 1:** Food Groups for Dietary Pattern Analysis.

No	Foods or Food Groups	Food Items
1	White rice	Cooked rice (well milled)
2	Multi-grain rice	Cooked rice with soybean, cooked rice with other cereals, half and half cooked rice well-milled and rice with soybean, half and half cooked rice well-milled and rice with other cereals
3	Noodles dumplings	Ramen, wheat noodles with soup, Chajangmyon/Jambbong, buckwheat vermicelli/buckwheat noodle, dumpling/dumpling with soup, starch vermicelli
4	Rice cake	Rice cake (plain rod shape)/rice cake with soup, other rice cakes
5	Bread	Loaf bread/sandwich/toast, jam/honey/butter/margarine, bread with small red bean, other breads
6	Pizza hamburger	Pizza/hamburger
7	Roasted grain powder	Parched cereal powder
8	Cereals and snacks	Cereals (corn flakes), cakes/Chocopie, cookie/cracker/snack, candy/chocolate
9	Potatoes and Sweet potatoes	Potatoes, sweet potatoes
10	Legumes and soybean paste	Legumes, soup and stew with soybean paste/soybean paste
11	Tofu	Tofu
12	Starch jelly	Starch jelly
13	Nuts	Nuts
14	Kimchi	Kimchi (Korean cabbage), Kkakduki/small radish kimchi, kimchi (radish with water), other kimchies(green onion/Kodulbbagi/mustard leaves)
15	Pickled vegetables	Korean style pickles
16	Yellow-green vegetable	Radish/Salted radish, Korean cabbages/Korean cabbage soup, spinach, lettuce, perilla leaf, vegetables wrap/vegetable salad, other green vegetables, pepper leaves/Chamnamul/Asterscaber, crown daisy/leek/water dropwort, cucumber, carrot/carrot juice, green pepper
17	Other vegetables	Deoduck/Doraji(kinds of white root), bean sprouts, bracken/sweet potato stalk/stem of taro, onion, pumpkin (immature), pumpkin (mature)/pumpkin juice
18	Mushrooms	Oyster mushroom, other mushrooms
19	Fruits and fruits juices	Strawberry, muskmelon/melon, watermelon, peach/plum, banana, persimmon (hard/dried), tangerine, pear/pear juice, apple/apple juice, orange/orange juice, grape/grape juice, tomato/cherry tomato/tomato juice
20	Red meat	Pork (belly), pork (roasted), pork (braised), steak/beef (roasted), dog meat, beef soup, beef soup with vegetables
21	Red meat byproducts	Edible viscera
22	Poultry	Fried chicken/chicken stew
23	Eggs	Eggs
24	Lean fish	Sashimi, hair tail, Eel, yellow croaker/sea bream/flat fish, Alaska pollack
25	Fatty fish	Mackerel/Pacific saury/Spanish mackerel
26	Shellfish	Clam/whelk, oyster
27	Other seafood	Cuttlefish/octopus, crab, shrimp
28	Dried anchovy	Dried anchovy
29	Salt fermented fish	Salt-fermented fish
30	Processed meats and fishes	Ham/sausage, tuna (canned), fish paste/crab flavored
31	Seaweeds	Laver (dried), kelp/sea mustard
32	Milk	Milk
33	Dairy products	Yogurt, ice cream, cheese
34	Carbonated beverage	Carbonated drinks
35	Coffee	Coffee, coffee sugar, coffee cream
36	Tea	Green tea
37	Other drinks	Soybean milk, other drinks

**Table 2:** Rotated Factor Loadings for Dietary Patterns Identified by Factor Analysis ^a.^

	Factor 1	Factor 2	Factor 3
	Meat Dietary Pattern	White Rice Dietary Pattern	Other Pattern
White rice		0.78721	
Multi-grain rice		−0.73148	
Noodles dumplings	0.47276		
Rice cake	0.35163		
Bread	0.58647		
Pizza hamburger	0.52652		
Roasted grain powder			
Cereals and snacks	0.57236		
Potatoes and Sweet potatoes		−0.208	0.38359
Legumes and soybean paste		−0.2104	0.44545
Tofu		−0.20417	0.44552
Starch jelly			0.27955
Nuts		−0.2836	
Kimchi			0.44016
Pickled vegetables			0.34359
Yellow-green vegetable			0.73465
Other vegetables			0.70435
Mushrooms			0.56969
Fruits and fruits juices			0.3369
Red meat	0.44857		0.25373
Red meat by-products	0.35232		
Poultry	0.43801		
Eggs	0.29667		0.20619
Lean fish			0.58239
Fatty fish			0.51097
Shellfish			0.43758
Other seafood	0.21544		0.41895
Dried anchovy			0.49245
Salt fermented fish		0.21363	0.28833
Processed meats and fishes	0.44043		0.23669
Seaweeds			0.56816
Milk		−0.2114	0.20504
Dairy products	0.21667		0.22627
Carbonated beverage			
Coffee			
Tea	0.26047		
Other beverage			

^a^ Absolute values greater than 0.2 were presented.

**Table 3:** Characteristics of Participants According to Quartiles of Each Dietary Pattern.

		Meat Dietary Pattern			
	**Total**	**Quartile 1**	**Quartile 2**	**Quartile 3**	**Quartile 4**
No. of participants	78320	19580	19580	19580	19580
Age (years)	52.3 (7.8)	55.7 (7.2)	53.4 (7.5)	51.3 (7.5)	49.0 (7.3)
Body mass index (kg/m^2^)	23.6 (2.9)	23.9 (2.9)	23.7 (2.9)	23.5 (2.9)	23.4 (3.0)
Education, Undergraduate school or more %	19.67	9.8	15.5	22.4	31.0
Marital status, Married or cohabitanting %	86.51	84.7	86.2	87.3	87.8
Regular physical activity, %	50.71	50.3	51.2	51.8	49.6
Drinking, Ever, %	32.71	23.5	31.1	35.9	40.3
Smoking, Ever, %	3.59	2.6	3.4	3.8	4.6
Total energy intake (kcal/day)	1981.0 (487.6)	1465.5 (394.0)	1522.2 (395.6)	1680.0 (414.0)	2056.5 (505.2)
				**White Rice Dietary Pattern**	
Age (years)	52.3 (7.8)	52.5 (7.7)	52.8 (7.8)	52.6 (7.8)	51.6 (7.8)
Body mass index (kg/m^2^)	23.6 (2.9)	23.5 (2.9)	23.7 (2.9)	23.7 (2.9)	23.7 (3.0)
Education, Undergraduate school or more %	19.67	23.6	18.9	18.5	17.7
Marital status, Married or cohabitanting %	86.51	87.0	86.8	86.8	85.5
Regular physical activity, %	50.71	58.4	52.7	49.6	42.2
Drinking, Ever, %	32.71	29.1	30.4	34.2	37.2
Smoking, Ever, %	3.59	2.5	2.5	3.8	5.6
Total energy intake (kcal/day)	1981.0 (487.6)	2044.4 (431.5)	1683.7 (347.9)	1469.7 (418.5)	1526.5 (517.7)
				**Other Pattern**	
Age (years)	52.3 (7.8)	51.7 (8.0)	52.5 (7.9)	52.6 (7.7)	52.73 (7.5)
Body mass index (kg/m^2^)	23.6 (2.9)	23.4 (3.0)	23.6 (2.9)	23.7 (2.9)	23.77 (2.9)
Education, Undergraduate school or more %	19.67	20.6	19.0	19.3	19.8
Marital status, Married or cohabitanting %	86.51	82.6	85.8	88.3	89.3
Regular physical activity, %	50.71	44.3	48.6	52.7	57.2
Drinking, Ever, %	32.71	33.7	32.3	32.6	32.3
Smoking, Ever, %	3.59	4.0	3.5	3.4	3.5
Total energy intake (kcal/day)	1981.0 (487.6)	1440.3 (440.7)	1569.9 (402.7)	1717.0 (421.1)	1997.0 (495.8)

^a^ Continuous variables are reported as mean (standard deviation) value and categorical variables are reported as %; ^b^ Analysis of variance was used for continuous variables and chi-square test was used for categorical variables.

**Table 4:** Hazard Ratios (HR) and 95% Confidence Intervals (CI) of Breast Cancer according to of Each Dietary Pattern Score.

	**Meat Dietary Pattern**				
	**Q1**	**Q2**	**Q3**	**Q4**	**P for Trend ^b^**
Person-years	126615.52	123501.63	122526.1	121847.07	
Cases	91	66	96	106	
HR (95% CI) ^a^	1.00 (reference)	0.70 (0.51, 0.97)	0.99 (0.73, 1.33)	1.05 (0.76, 1.47)	0.3047
	**White Rice Dietary Pattern**				
	**Q1**	**Q2**	**Q3**	**Q4**	**P for Trend ^b^**
Person-years	122953.56	122874.99	123006.34	125655.42	
Cases	88	90	76	105	
HR (95% CI) ^a^	1.00 (reference)	1.14 (0.84, 1.55)	1.00 (0.72, 1.40)	1.35 (1.00, 1.84)	0.0384
	**Other Pattern**				
	**Q1**	**Q2**	**Q3**	**Q4**	**P for Trend ^b^**
Person-years	121922.55	121161.98	123307.24	128098.56	
Cases	74	84	92	109	
HR (95% CI) ^a^	1.00 (reference)	1.12 (0.82, 1.54)	1.18 (0.86, 1.61)	1.30 (0.94, 1.80)	0.1222

^a^ HR (95% CI): adjusted for BMI (kg/m^2^, continuous), total energy intake (kcal/day, continuous), educational level (middle school or less, high school or college, undergraduate or more), parity (nulliparous, 1, 2, ≥3), age at first birth (nulliparous, aged <25 years at first birth, aged ≥25 at first birth), age at menarche (<15, 15, ≥16 years), regular exercise (no, yes), alcohol intake (never, ever), and the presence of a family history of breast cancer (no, yes); ^b^ P for trend was calculated using the median value of each category as a continuous variable.

**Table 5:** Hazard Ratios (HR) and 95% Confidence intervals (CI) of Breast Cancer according to White Rice Consumption.

	White rice Consumption (Servings)			
	< 1/day	1–2/day	3/day ≤	P for Trend ^b^
**Total**				
Number	65265	5080	7975	
Breast Cancer Cases	291	23	45	
Person-year	410894.11	32071.06	51525.15	
HR (95% CI) ^a^	1.00 (reference)	1.07 (0.70, 1.65)	1.29 (0.94, 1.76)	0.1363
**Age < 50 years**				
Number	23964	2515	3324	
Breast Cancer Cases	118	10	22	
Person-year	154998.93	16160.12	21877.67	
HR (95% CI) ^a^	1.00 (reference)	0.86 (0.45, 1.66)	1.39 (0.88, 2.20)	0.2945
**Age ≥ 50 years**				
Number	41301	2565	4651	
Breast Cancer Cases	173	13	23	
Person-year	255895.18	15910.94	29647.48	
HR (95% CI) ^a^	1.00 (reference)	1.30 (0.73, 2.32)	1.20 (0.77, 1.85)	0.2876

^a^ HR (95% CI): adjusted for BMI (kg/m^2^, continuous), energy intake (kcal/day, continuous), education level (middle school or less, high school or college, undergraduate or more), parity (nulliparous, 1, 2, more than three), age at first birth (nulliparous, aged <25 years at first birth, aged ≥25 at first birth), age at menarche (<15, 15, ≥16 years), oral contraceptive use (never, ever), regular exercise (no, yes), alcohol intake (never, ever), and the presence of a family history of breast cancer (no, yes); ^b^ P for trend was calculated using the median value of each category as a continuous variable.

**Table 6:** Hazard Ratios (HR) and 95% Confidence Intervals (CI) of Breast Cancer according to Multi-Grain Rice Consumption.

	Multi-Grain Rice Consumption (Sservings)			
	≤ 1/day	2/day	3/day ≤	P for Trend ^b^
**Total**				
Number	36843	14385	27092	
Breast Cancer Cases	178	62	119	
Person-year	237597.61	87197.55	169695.16	
HR (95% CI) ^a^	1.00 (reference)	0.96 (0.71, 1.26)	0.89 (0.70, 1.14)	0.3463
**Age < 50 years**				
Number	15246	5311	9246	
Breast Cancer Cases	91	20	39	
Person-year	100309.15	33014.31	59713.25	
HR (95% CI) ^a^	1.00 (reference)	0.66 (0.41, 1.08)	0.67 (0.45, 0.99)	0.0204
**Age ≥ 50 years**				
Number	21597	9074	17846	
Breast Cancer Cases	87	42	80	
Person-year	137288.46	54183.24	109981.9	
HR (95% CI) ^a^	1.00 (reference)	1.22 (0.84, 1.77)	1.09 (0.79, 1.50)	0.4602

^a^ HR (95% CI): adjusted for BMI (kg/m^2^, continuous), energy intake (kcal/day, continuous), education level (middle school or less, high school or college, undergraduate or more), parity (nulliparous, 1, 2, more than three), age at first birth (nulliparous, aged <25 years at first birth, aged ≥25 at first birth), age at menarche (<15, 15, ≥16 years), oral contraceptive use (never, ever), regular exercise (no, yes), alcohol intake (never, ever), and the presence of a family history of breast cancer (no, yes); ^b^ P for trend was calculated using the median value of each category as a continuous variable. No association was observed for the meat dietary pattern and the other pattern.

## References

[1] Bray F., Ferlay J., Soerjomataram I., Siegel R.L., Torre L.A., Jemal A. (2018). Global cancer statistics 2018: GLOBOCAN estimates of incidence and mortality worldwide for 36 cancers in 185 countries. CA Cancer J. Clin..

[2] Jung K.W., Won Y.J., Kong H.J., Lee E.S. (2019). Cancer Statistics in Korea: Incidence, Mortality, Survival, and Prevalence in 2016. Cancer Res. Treat..

[3] Smith-Warner S.A., Spiegelman D., Yaun S.S., Adami H.O., Beeson W.L., Van Den Brandt P.A., Folsom A.R., Fraser G.E., Freudenheim J.L., Goldbohm R.A. (2001). Intake of fruits and vegetables and risk of breast cancer: A pooled analysis of cohort studies. JAMA.

[4] Wakai K., Tamakoshi K., Date C., Fukui M., Suzuki S., Lin Y., Niwa Y., Nishio K., Yatsuya H., Kondo T. (2005). Dietary intakes of fat and fatty acids and risk of breast cancer: A prospective study in Japan. Cancer Sci..

[5] Cho E., Chen W.Y., Hunter D.J., Stampfer M.J., Colditz G.A., Hankinson S.E., Willett W.C. (2006). Red meat intake and risk of breast cancer among premenopausal women. Arch. Intern. Med..

[6] Shin W.K., Lee H.W., Shin A., Lee J.K., Kang D. (2020). Milk Consumption Decreases Risk for Breast Cancer in Korean Women under 50 Years of Age: Results from the Health Examinees Study. Nutrients.

[7] Holmes M.D., Colditz G.A., Hunter D.J., Hankinson S.E., Rosner B., Speizer F.E., Willett W.C. (2003). Meat, fish and egg intake and risk of breast cancer. Int. J. Cancer.

[8] Colacurci N., De Franciscis P., Atlante M., Mancino P., Monti M., Volpini G., Benvenuti C. (2013). Endometrial, breast and liver safety of soy isoflavones plus Lactobacillus sporogenes in post-menopausal women. Gynecol. Endocrinol..

[9] Chajes V., Romieu I. (2014). Nutrition and breast cancer. Maturitas.

[10] Freudenheim J.L., Marshall J.R., Vena J.E., Laughlin R., Brasure J.R., Swanson M.K., Nemoto T., Graham S. (1996). Premenopausal breast cancer risk and intake of vegetables, fruits, and related nutrients. J. Natl. Cancer Inst..

[11] Hu F.B. (2002). Dietary pattern analysis: A new direction in nutritional epidemiology. Curr. Opin. Lipidol..

[12] Sieri S., Krogh V., Pala V., Muti P., Micheli A., Evangelista A., Tagliabue G., Berrino F. (2004). Dietary patterns and risk of breast cancer in the ORDET cohort. Cancer Epidemiol. Biomarkers Prev..

[13] Link L.B., Canchola A.J., Bernstein L., Clarke C.A., Stram D.O., Ursin G., Horn-Ross P.L. (2013). Dietary patterns and breast cancer risk in the California Teachers Study cohort. Am. J. Clin. Nutri..

[14] Adebamowo C.A., Hu F.B., Cho E., Spiegelman D., Holmes M.D., Willett W.C. (2005). Dietary patterns and the risk of breast cancer. Ann. Epidemiol..

[15] Fung T.T., Hu F.B., Holmes M.D., Rosner B.A., Hunter D.J., Colditz G.A., Willett W.C. (2005). Dietary patterns and the risk of postmenopausal breast cancer. Int. J. Cancer..

[16] Cottet V., Touvier M., Fournier A., Touillaud M.S., Lafay L., Clavel-Chapelon F., Boutron-Ruault M.-C. (2009). Postmenopausal breast cancer risk and dietary patterns in the E3N-EPIC prospective cohort study. Am. J. Epidemiol..

[17] Terry P., Suzuki R., Hu F.B., Wolk A. (2001). A prospective study of major dietary patterns and the risk of breast cancer. Cancer Epidemiol. Biomarkers Prev..

[18] Kim S., Moon S., Popkin B.M. (2000). The nutrition transition in South Korea. Am. J. Clin. Nutri..

[19] Daniel C.R., Cross A.J., Koebnick C., Sinha R. (2011). Trends in meat consumption in the USA. Public Health Nutr..

[20] Cui X., Dai Q., Tseng M., Shu X.-O., Gao Y.T., Zheng W. (2007). Dietary patterns and breast cancer risk in the shanghai breast cancer study. Cancer Epidemiol. Biomarkers Prev..

[21] Kojima R., Okada E., Ukawa S., Mori M., Wakai K., Date C., Iso H., Tamakoshi A. (2017). Dietary patterns and breast cancer risk in a prospective Japanese study. Breast Cancer.

[22] Cho Y.A., Kim J., Shin A., Park K.S., Ro J. (2010). Dietary patterns and breast cancer risk in Korean women. Nutr. Cancer.

[23] Health Examinees (HEXA) Study Group (2015). The Health Examinees (HEXA) study: Rationale, study design and baseline characteristics. Asian Pac. J. Cancer Prev..

[24] Kim Y., Han B.G., Koges Group (2016). Cohort profile: The Korean genome and epidemiology study (KoGES) consortium. Int. J. Epidemiol..

[25] Shin S., Lee H.W., Kim C., Lim J., Lee J.K., Lee S.A., Kang D. (2017). Egg consumption and risk of metabolic syndrome in Korean adults: Results from the Health Examinees Study. Nutrients.

[26] Ahn Y., Kwon E., Shim J., Park M., Joo Y., Kimm K., Park C., Kim D. (2007). Validation and reproducibility of food frequency questionnaire for Korean genome epidemiologic study. Eur. J. Clin. Nutr..

[27] KCDC Center for Genome Sciences FFQ Guideline for Korean Genome and Epidemiology Study (KoGES) 2014. http://www.nih.go.kr/menu.es?mid=a40504100200.

[28] Korean Nutrition Society (2000). Food Composition Table. Recommended Dietary Allowances for Koreans.

[29] Choi J.H., Woo H.D., Lee J.H., Kim J. (2015). Dietary patterns and risk for metabolic syndrome in Korean women: A cross-sectional study. Medicine.

[30] Song S., Kim J., Kim J. (2018). Gender differences in the association between dietary pattern and the incidence of hypertension in middle-aged and older adults. Nutrients.

[31] Lee K.W., Woo H.D., Cho M.J., Park J.K., Kim S.S. (2019). Identification of dietary patterns associated with incidence of hyperglycemia in middle-aged and older Korean adults. Nutrients.

[32] Agurs-Collins T., Rosenberg L., Makambi K., Palmer J.R., Adams-Campbell L. (2009). Dietary patterns and breast cancer risk in women participating in the Black Women’s Health Study. Am. J. Clin. Nutr..

[33] Shin S., Saito E., Inoue M., Sawada N., Ishihara J., Takachi R., Nanri A., Shimazu T., Yamaji T., Iwasaki M. (2016). Dietary pattern and breast cancer risk in Japanese women: The Japan Public Health Center-based Prospective Study (JPHC Study). Br. J. Nutr..

[34] Butler L.M., Wu A.H., Wang R., Koh W.P., Yuan J.M., Yu M.C. (2010). A vegetable-fruit-soy dietary pattern protects against breast cancer among postmenopausal Singapore Chinese women. Am. J. Clin. Nutri..

[35] Steffen L.M., Jacobs Jr D.R., Stevens J., Shahar E., Carithers T., Folsom A.R. (2003). Associations of whole-grain, refined-grain, and fruit and vegetable consumption with risks of all-cause mortality and incident coronary artery disease and ischemic stroke: The Atherosclerosis Risk in Communities (ARIC) Study. Am. J. Clin. Nutr..

[36] Kim J., Lee Y., Lee S.Y., Kim Y.O., Chung Y.-S., Park S.B. (2013). Dietary patterns and functional disability in older Korean adults. Maturitas.

[37] Yun S.H., Kim K., Nam S.J., Kong G., Kim M.K. (2010). The association of carbohydrate intake, glycemic load, glycemic index, and selected rice foods with breast cancer risk: A case-control study in South Korea. Asia Pac. J. Clin. Nutr..

[38] Augustin L., Dal Maso L., La Vecchia C., Parpinel M., Negri E., Vaccarella S., Kendall C., Jenkins D., Franceschi S. (2001). Dietary glycemic index and glycemic load, and breast cancer risk: A case-control study. Ann. Oncol..

[39] Woo H.D., Park K.S., Shin A., Ro J., Kim J. (2013). Glycemic index and glycemic load dietary patterns and the associated risk of breast cancer: A case-control study. Asian Pac. J. Cancer Prev..

[40] Sonestedt E., Borgquist S., Ericson U., Gullberg B., Landberg G., Olsson H., Wirfält E. (2008). Plant foods and oestrogen receptor α-and β-defined breast cancer: Observations from the Malmö Diet and Cancer cohort. Carcinogenesis.

[41] Tajaddini A., Pourzand A., Sanaat Z., Pirouzpanah S. (2015). Dietary resistant starch contained foods and breast cancer risk: A case-control study in northwest of Iran. Asian Pac. J. Cancer Prev..

[42] (2014). Ministry of Health and Welfare, Korea Centers for Disease Control and Prevention. Korea Health Statistics 2013: Korea National Health and Nutrition Examination Survey (NHANES VI-1).

[43] Lee H.P., Gourley L., Duffy S.W., Estève J., Lee J., Day N.E. (1992). Risk factors for breast cancer by age and menopausal status: A case-control study in Singapore. Cancer Cause Control.

[44] Meyskens J.F. (1992). Strategies for prevention of cancer in humans. Oncology (Williston Park N.Y.).

[45] Slavin J., Jacobs D., Marquart L. (1997). Whole-grain consumption and chronic disease: Protective mechanisms. Nutri. Cancer.

[46] Ahn Y., Park S.J., Kwack H., Kim M.K., Ko K.P., Kim S.S. (2013). Rice-eating pattern and the risk of metabolic syndrome especially waist circumference in Korean Genome and Epidemiology Study (KoGES). BMC Public Health.

[47] Slavin J.L. (1994). Epidemiological evidence for the impact of whole grains on health. Crit. Rev. Food Sci. Nutr..

[48] Cohen L.A., Zhao Z., Zang E.A., Wynn T.T., Simi B., Rivenson A. (1996). Wheat Bran and Psylium Diets: Effects on N-Methylnitrosourea-Induced Mammary Tumorigenesis in F344 Rats. J. Natl. Cancer Inst..

[49] Aune D., Chan D., Greenwood D., Vieira A., Rosenblatt D.N., Vieira R., Norat T. (2012). Dietary fiber and breast cancer risk: A systematic review and meta-analysis of prospective studies. Ann. Oncol..

[50] Wang D., Chuang H.C., Weng S.C., Huang P.H., Hsieh H.Y., Kulp S.K., Chen C.S. (2009). α-Tocopheryl succinate as a scaffold to develop potent inhibitors of breast cancer cell adhesion. J. Med. Chem..

[51] Mazur W., Adlercreutz H. (1998). Natural and anthropogenic environmental oestrogens: The scientific basis for risk assessment. Pure Appl. Chem..

[52] Brzezinski A., Debi A. (1999). Phytoestrogens: The “natural” selective estrogen receptor modulators?. Eur. J. Obstet. Gynecol. Reprod. Biol..

[53] Jung K.W., Won Y.J., Oh C.M., Kong H.J., Lee D.H., Lee K.H. (2017). Cancer statistics in Korea: Incidence, mortality, survival, and prevalence in 2014. Cancer Res. Treat..

[54] Cho S., Shin A., Song D., Park J.K., Kim Y., Choi J.Y., Kang D., Lee J.K. (2017). Validity of self-reported cancer history in the health examinees (HEXA) study: A comparison of self-report and cancer registry records. Cancer Epidemiol..

[55] Hebert J.R., Miller D.R. (1988). Methodologic considerations for investigating the diet-cancer link. Am. J. Clin. Nutri..

[56] Baglietto L., Krishnan K., Severi G., Hodge A., Brinkman M., English D., McLean C., Hopper J., Giles G. (2011). Dietary patterns and risk of breast cancer. Br. J. Cancer.

[57] Cho Y., Kim J., Park K., Lim S., Shin A., Sung M., Ro J. (2010). Effect of dietary soy intake on breast cancer risk according to menopause and hormone receptor status. Eur. J. Clin. Nutr..

[58] Martinez M.E., Marshall J.R., Sechrest L. (1998). Invited commentary: Factor analysis and the search for objectivity. Am. J. Epidemiol..

